# Procedural Sedation with Dexmedetomidine for Anterior Mediastinotomy in a High-Risk Patient

**DOI:** 10.1155/2022/3519003

**Published:** 2022-02-21

**Authors:** Miguel Pratas, Jorge Aires, Nuno Pereira da Silva, Tiago Oliveira, Cristovão Pinto, Jiele Li, Ana Filipa Ribeiro

**Affiliations:** Centro Hospitalar e Universitário de Coimbra, Coimbra, Portugal

## Abstract

Perioperative management of patients with mediastinal masses still poses a challenge for the anesthesiologist, as the use of general anesthesia can be associated with acute perioperative cardiorespiratory impairment resulting from the mass collapsing on the airway or vascular structures. Dexmedetomidine can be used for procedural sedation due to its reversible sedative and anxiolytic properties with dose-dependent effects, while not interfering with ventilatory drive. These features are of particular interest for the perioperative management of patients with large anterior mediastinal masses. In this case, we report our anesthetic management of a 22-year-old male scheduled for anterior mediastinotomy, with a large anterior mediastinal mass, with 50% distal tracheal compression and marked collapse of the superior vena cava and brachiocephalic trunk. In the operation theatre, an infusion of dexmedetomidine was titrated to adequate anesthetic depth while keeping the patient under spontaneous ventilation with oxygen (O_2_) supplementation and local anesthetic infiltration of the surgical site. Mediastinotomy lasted for about 30 minutes, during which the patient maintained appropriate ventilation and hemodynamic stability. No adverse events occurred perioperatively. Diagnostic procedures such as mediastinotomy for tissue biopsy are necessary to achieve a histological diagnosis. High-risk patients may present with severe postural symptoms, stridor, cyanosis, and radiological evidence of more than 50% airway obstruction, tracheal compression with bronchial compression, pericardial effusion, or superior vena cava syndrome. Relaxation of bronchial smooth muscles under general anesthesia increases the risk of airway obstruction. In this case, with the use of dexmedetomidine combined with local anesthetic infiltration, spontaneous ventilation and muscle tone were preserved, decreasing the probability of intraoperative complications. It is our opinion that dexmedetomidine combined with local anesthetic infiltration can be a safe option for procedural sedation in patients presenting with high-risk anterior mediastinal masses for mediastinotomy.

## 1. Introduction

Mediastinal masses are an uncommon entity. They constitute a heterogeneous group of benign and malignant tumors [1].

Location and size of the mediastinal tumor may vary, such as clinical presentation—ranging from a complete lack of symptoms to severe cardiorespiratory problems [2].

Perioperative management of patients with mediastinal masses still poses a challenge for the anesthesiologist as the use of general anesthesia can be associated with acute perioperative cardiorespiratory compromise resulting from the mass collapsing on the trachea, cardiac chambers, pulmonary veins, or the superior vena cava [3].

Dexmedetomidine is an alpha2-adrenergic agonist that can be used for procedural sedation due to its reversible sedative and anxiolytic properties with dose-dependent effects, ranging from minimal to deep sedation and the benefit of not impairing the ventilatory drive [4]. These unique features can be particularly useful in patients presenting with mediastinal masses.

Literature is scarce on the use of dexmedetomidine for procedural sedation in this setting.

This report aims to detail anesthetic management using dexmedetomidine in a patient with a large anterior mediastinal mass scheduled for anterior mediastinotomy.

## 2. Case Presentation

**Figure 1:**
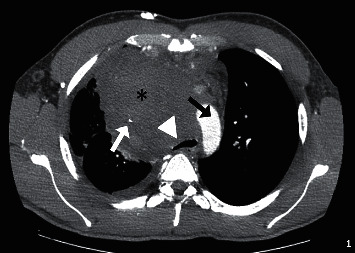
Contrast-enhanced computer tomography (CECT) with axial reformat shows a bulky anterior mediastinal mass (black asterisk) encasing and compressing the superior vena cava (white arrow), resulting in superior vena cava syndrome. The mass also deviates from the aortic arch (black arrow) and the trachea (white arrowhead), with a marked reduction of the tracheal diameter.

**Figure 2:**
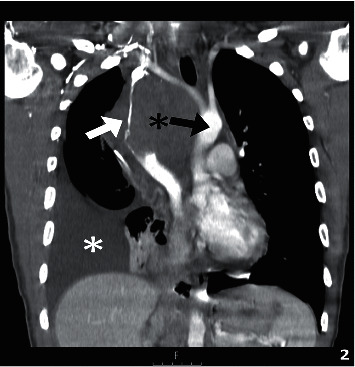
Contrast-enhanced computer tomography (CECT) with coronal reformat shows a bulky anterior mediastinal mass (black asterisk) encasing and compressing the superior vena cava (white arrow), resulting in superior vena cava syndrome. The mass also deviates from the aortic arch (black arrow), and a moderate pleural effusion is also present (white asterisk).

**Figure 3:**
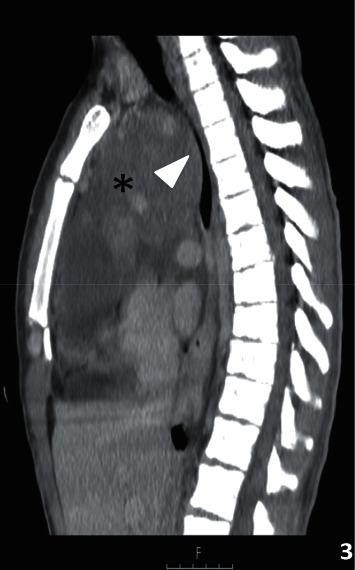
Contrast-enhanced computer tomography (CECT) with sagittal reformat shows a bulky anterior mediastinal mass (black asterisk). The mass also deviates from the aortic arch and the trachea (white arrowhead), with a marked reduction of the tracheal diameter.

A 22-year-old male patient with no relevant past medical history and an unremarkable family history was admitted to the hospital with complaints of worsening dry cough, dyspnea on exertion, and occasional night fever. No abnormal findings were noted on his physical examination. During admission, he underwent a thoracic computer tomography scan which revealed a large expansive heterogeneous mass in the anterior mediastinum measuring 16.1 × 10.4 × 17.5 cm causing mediastinum shift, obstruction (50%) of the distal third of the trachea, and marked compression of the superior vena cava and brachiocephalic trunk. Extensive collateral circulation in the thoracic wall and a large right pleural effusion were also noted (Figures 1–3).

The patient was transferred to the cardiothoracic surgery ward for elective anterior mediastinotomy for multiple biopsies of the mediastinal mass.

On the preoperative assessment, the patient was conscious and oriented to time, person, and space, presenting mild dyspnea with efforts, a peripheral oxygen saturation of 95% on room air, and no clinical signs of superior vena cava syndrome despite imagological findings. The patient was classified by the American Society of Anesthesiology Physical Status (ASA-PS) classification system as ASA-PS 1.

Additional preoperative testing included electrocardiogram (ECG), complete blood count, coagulation studies, renal and liver function tests, and a transthoracic echocardiogram, all of which were unremarkable to the anesthetic plan.

Prior to the procedure, a right thoracic drain was placed in order to relieve the pleural effusion. Premedication with 5 mg of intramuscular midazolam was given 1 hour before the surgery.

In the operating room, the patient was monitored according to the standard American Society of Anesthesiology (ASA) guidelines with additional Bispectral Index monitoring (BIS) and invasive arterial blood pressure. Two peripheral high bore intravenous catheters in the lower limbs and a right side radial arterial line were placed.

Ready availability of rigid bronchoscopy was arranged, and the cardiopulmonary bypass team was put on standby.

A bolus dose of 0.2 mg/kg of intravenous ketamine was given and an infusion of dexmedetomidine was started at 0.7 mcg/kg/min and titrated to a BIS value of 60, while keeping the patient under spontaneous ventilation with O_2_ supplementation by nasal cannula (inspired fraction of oxygen of 31%). Approximately 10 minutes after starting the infusion, local anesthetic infiltration of the surgical site with 2% lidocaine was performed by the surgeon.

Mediastinotomy lasted for about 30 minutes, during which the patient maintained spontaneous ventilation and hemodynamic stability. Dexmedetomidine infusion was stopped at the end of the procedure and within 5 minutes the patient regained conscience and was able to follow simple orders without reporting pain or awareness during the surgery.

No adverse events occurred perioperatively.

## 3. Discussion

Anesthesia for biopsy or excision of mediastinal masses is associated with a high risk of severe airway obstruction, hemodynamic compromise, and death [5].

Diagnostic procedures such as mediastinoscopy or mediastinotomy for tissue biopsy are necessary to achieve a histological diagnosis and therefore establish the course of treatment.

The presence of respiratory symptoms such as dyspnea, orthopnea, postural dyspnea, or stridor must alert for central airway compression and increased risk of perioperative complications [6]. Patients can be categorized into three categories according to risk:

- Low risk
- Middle risk
- High risk

Those considered to be high risk may present with severe postural symptoms, stridor, cyanosis, and radiological evidence of more than 50% airway obstruction, tracheal compression with bronchial compression, pericardial effusion, or superior vena cava syndrome [6]. Relaxation of bronchial smooth muscles under anesthesia increases the risk of airway obstruction since they are already made narrow by the external pressure of the mass [7]. Awake fiberoptic intubation, maintenance of spontaneous ventilation, avoidance of muscle relaxants, intubation distal to the airway compression, positioning changes, and immediate availability of rigid bronchoscopy can be useful strategies regarding the anesthetic management of these patients [1].

Cardiopulmonary bypass can be considered in situations when the ability to ventilate is unlikely or uncertain after induction, e.g., if the tumor compresses the distal third of the trachea, both mainstem bronchi, or the carina [1].

Dexmedetomidine (an alpha2-adrenergic agonist) is an antihypertensive agent that can also be used for procedural sedation. It has rapidly reversible sedative and anxiolytic properties with dose-dependent effects, ranging from minimal to deep sedation. Dexmedetomidine does not impair the respiratory drive [4], and sedation is characterized by preserved muscle tone and ventilation, by spontaneous and evoked movements, and awakening to external stimuli [8].

We considered this case to be a high-risk patient. As such, preserving spontaneous ventilation and muscle tone in order to decrease the probability of intraoperative complications was of paramount importance. The use of dexmedetomidine and ketamine has proven their usefulness due to their safety profile, with analgesic and sedative properties but a low risk of respiratory depression [9]. Additional analgesia was provided by infiltration with local anesthetics. Nevertheless, alternative options such as rigid bronchoscopy and cardiopulmonary bypass should be carefully planned as an alternative approach and be readily available.

It is our opinion that dexmedetomidine combined with local anesthetic infiltration could be a safe option for procedural sedation in patients presenting with high-risk anterior mediastinal masses for mediastinotomy. Careful planning and alternative approaches to anesthetic management should be readily available.

## Abbreviations

ASA: American Society of Anesthesiology
ASA-PS: American Society of Anesthesiology Physical Status
BIS: Bispectral index
CT: Computer tomography
ECG: Electrocardiogram
O_2_: Oxygen.
